# Partial trisomy 21 map: Ten cases further supporting the highly restricted Down syndrome critical region (HR‐DSCR) on human chromosome 21

**DOI:** 10.1002/mgg3.797

**Published:** 2019-06-25

**Authors:** Maria Chiara Pelleri, Elena Cicchini, Michael B. Petersen, Lisbeth Tranebjærg, Teresa Mattina, Pamela Magini, Francesca Antonaros, Maria Caracausi, Lorenza Vitale, Chiara Locatelli, Marco Seri, Pierluigi Strippoli, Allison Piovesan, Guido Cocchi

**Affiliations:** ^1^ Department of Experimental, Diagnostic and Specialty Medicine (DIMES), Unit of Histology, Embryology and Applied Biology University of Bologna Bologna (BO) Italy; ^2^ Department of Genetics Aalborg University Hospital Aalborg Denmark; ^3^ Department of Clinical Genetics Aalborg University Aalborg Denmark; ^4^ Department of Clinical Genetics/Rigshospitalet The Kennedy Centre Glostrup Denmark; ^5^ University of Copenhagen, Institute of Clinical Medicine, The Panum Institute Copenhagen N Denmark; ^6^ Department of Pediatrics Medical Genetics University of Catania Italy; ^7^ Medical Genetics Unit St. Orsola‐Malpighi Polyclinic Bologna (BO) Italy; ^8^ Neonatology Unit St. Orsola‐Malpighi Polyclinic Bologna (BO) Italy; ^9^ Medical Genetics Unit, Department of Medical and Surgical Sciences (DIMEC) St. Orsola‐Malpighi Polyclinic, University of Bologna Bologna (BO) Italy; ^10^ Neonatology Unit, Department of Medical and Surgical Sciences (DIMEC) St. Orsola‐Malpighi Polyclinic, University of Bologna Bologna (BO) Italy

**Keywords:** computational biology, Down syndrome, highly restricted Down syndrome critical region, human chromosome 21, intellectual disability, partial trisomy 21

## Abstract

**Background:**

Down syndrome (DS) is characterized by the presence of an extra full or partial human chromosome 21 (Hsa21). An invaluable model to define genotype‐phenotype correlations in DS is the study of the extremely rare cases of partial (segmental) trisomy 21 (PT21), the duplication of only a delimited region of Hsa21 associated or not to DS. A systematic retrospective reanalysis of 125 PT21 cases described up to 2015 allowed the creation of the most comprehensive PT21 map and the identification of a 34‐kb highly restricted DS critical region (HR‐DSCR) as the minimal region whose duplication is shared by all PT21 subjects diagnosed with DS. We reanalyzed at higher resolution three cases previously published and we accurately searched for any new PT21 reports in order to verify whether HR‐DSCR limits could prospectively be confirmed and possibly refined.

**Methods:**

Hsa21 partial duplications of three PT21 subjects were refined by adding array‐based comparative genomic hybridization data. Seven newly described PT21 cases fulfilling stringent cytogenetic and clinical criteria have been incorporated into the PT21 integrated map.

**Results:**

The PT21 map now integrates fine structure of Hsa21 sequence intervals of 132 subjects onto a common framework fully consistent with the presence of a duplicated HR‐DSCR, on distal 21q22.13 sub‐band, only in DS subjects and not in non‐DS individuals. No documented exception to the HR‐DSCR model was found.

**Conclusions:**

The findings presented here further support the association of the HR‐DSCR with the diagnosis of DS, representing an unbiased validation of the original model. Further studies are needed to identify and characterize genetic determinants presumably located in the HR‐DSCR and functionally associated to the critical manifestations of DS.

## 1 INTRODUCTION

In 1959 Lejeune, Gautier, and Turpin showed that human chromosome 21 (Hsa21) is present in an extra copy (trisomy 21) in the cells of subjects with Down syndrome (DS, OMIM #190685) (Lejeune, Gauthier, & Turpin, 1959), the most common constitutional form of intellectual disability (ID) (Gardiner et al., 2010; Menghini, Costanzo, & Vicari, 2011; Tolksdorf & Wiedemann, 1981). Although Hsa21 has the smallest number of genes among the human autosomal chromosomes (Piovesan, Caracausi, Antonaros, Pelleri, & Vitale, 2016), it has been difficult to link the function of specific Hsa21 genes to distinct phenotypic features of subjects with DS, whose more constant manifestations are a typical facies (oblique eyes, flat nasal bridge) and ID, together with other variable signs and symptoms (Epstein, 1989; Gardiner et al., 2010; Hickey, Hickey, & Summar, 2012; Letourneau & Antonarakis, 2012; Megarbane et al., 2009; Roizen & Patterson, 2003; Strippoli et al., 2013).

An invaluable model for linking genotype and phenotype in DS is the study of the extremely rare cases of partial (segmental) trisomy 21 (PT21), the duplication of only a delimited segment of Hsa21 associated or not to DS. PT21 was first reported by Ilbery and coll. (Ilbery, Lee, & Winn, 1961) as "incomplete trisomy" and has been widely studied by methods with an increasing power of resolution in order to establish correlations between the gene content of the duplicated segment and the associated signs and symptoms (reviewed in (Pelleri et al., 2016)). These studies have strongly supported the concept that not all Hsa21 loci are required for the manifestation of DS, as anticipated by Lejeune who hypothesized the presence of a few "culprits among so many innocents" on Hsa21 because if most of the genes would produce harm when in triplicate, "trisomic children would not survive at all. Few of the accelerated reactions are dangerous" (Lejeune, 1990).

Following multiple case reports of individuals with PT21 in the 70s–90s, the concept of "Down syndrome critical region" (DSCR) arose, with different grades of support from different authors (reviewed in (Pelleri et al. 2016)). Systematic attempts were then published in 2009 to identify "critical regions" on Hsa21 for several distinct phenotypes observed in DS, exploiting the availability of a larger number of cases (Korbel et al., 2009; Lyle et al., 2009). We have recently proposed a systematic reanalysis of all described PT21 cases (from 1973 to 2015) by building an integrated, comparative map of 125 cases with or without DS fulfilling stringent cytogenetic and clinical criteria (Pelleri et al., 2016). An innovation was also the use of the diagnosis of DS as the phenotype to be mapped, thus focusing on the Hsa21 minimal duplicated region shared by all the subjects diagnosed with DS, whose most constant features are ID and some facial phenotypes, that is oblique eyes and flat nasal bridge. The thorough reanalysis and comparison of the data available over several decades required the correction and update of cytogenetic band boundaries as well as of relative order and exact position of many genomic markers, integration of analyses subsequently performed in the course of years on the same identifiable subjects even if reported by different authors, corrections of incongruencies in clinical classification or cytogenetic characterization, and removal of cases with any residual uncertainty in the presentation of data. This approach finally allowed the inclusion or exclusion of fine Hsa21 sequence intervals as candidates for DS, also integrating duplication copy number variants (CNVs) data. The main result was the identification of a highly restricted DSCR (HR‐DSCR) of only 34 kilobases (kb) on distal 21q22.13 as the minimal region whose duplication is shared by all DS subjects and is absent in all non‐DS subjects, containing no known gene and with relevant homology only to the chimpanzee genome (Pelleri et al., 2016).

When a similar systematic approach has been applied to congenital heart disease (CHD) in subjects with DS, a more distal region has been shown to be associated to CHD in DS, possibly with different limits according to the specific type of CHD, in the context of a multifactorial model (Pelleri et al., 2017). These findings further support the specificity of the results previously obtained for the HR‐DSCR that appears to be more proximal, well‐delimited, and monofactorial in its association to the diagnosis of DS.

Further studies are needed to confirm that HR‐DSCR is really functionally associated to the critical manifestations of DS, and in particular to ID. While research is in progress to identify functioning loci still unknown but possibly present in the HR‐DSCR, it is also fundamental to continue the study of any new cases of PT21 and to refine the limits of the trisomic Hsa21 portions in cases already studied at low resolution. The confirmation that there is no documented exception to the HR‐DSCR model would further encourage molecular studies in this small segment of Hsa21 as well as high resolution analysis to investigate some very interesting cases reported in 90s: they are five phenocopies (people with DS phenotype and no evidence of cytogenetic/molecular alteration) (Ahlbom et al., 1996; Anneren & Edman, 1993; McCormick et al., 1989) and the opposite condition of apparent full trisomy 21 without DS phenotype (Avramopoulos et al., 1997). No marker through high resolution techniques was analyzed within the HR‐DSCR, therefore the possibility that a very small Hsa21 region might be involved in the expression of basic DS features cannot be excluded.

Due to the rarity of these particular cases as well as of PT21 cases, an international collaborative effort would be desirable in order to perform cutting‐edge analysis and collect new data.

In this light, the aim of this study was to reanalyze at higher resolution three cases already included in the PT21 integrated map in order to verify whether this analysis could help in confirming and refining the limits of the HR‐DSCR. In addition, we accurately searched for any new reports of PT21 subsequent to the original study to integrate the new data in the existing PT21 map searching again for confirmation or rejection of the original model. This second approach is of particular relevance because, being a prospective study started after the first description of HR‐DSCR, it represents an unbiased validation of the original model that could also be performed in the future.

## 2 MATERIALS AND METHODS

### 2.1 Molecular cytogenetic characterization

In order to update the PT21 map (125 PT21 cases with or without DS) previously published (Pelleri et al., 2016), we reanalyzed at higher resolution three cases already included in the previous map. These three reanalyzed cases are numbered here from 1 to 3 (Table 1); the correspondence with previous map identifiers ("Map ID" in (Pelleri et al., 2016)) and with updated map identification numbers ("Map ID here") is reported in Table 1.

**Table 1:** Summarized data about partial trisomy 21 cases mapped here. Cases 1–3 were reanalyzed here, cases 4 and 7–10 were newly described and reviewed here, cases 5 and 6 were the two additionally retrieved cases. Sex and age: as in the first report about the subject; M: male; F: female. Map ID here: identification numbers as reported in Tables S1 and S2

Case no.	Subject	Diagnosis	Sex	Age	Karyotype	Method	Country	Reference	Map ID in Pelleri et al. (2016)	Map ID here
1	MP01	DS	M	6.5 yrs	46,XY,dup(21)(q22.1‐‐>qter) de novo	Banding Southern Blot Array‐CGH (here)	Denmark	McCormick et al. (1989), Petersen et al. (1990)	#043	#044
2	MP03	Non‐DS	F	25 yrs	46,XX,dup(21)(q11.2‐‐>q21.2)pat de novo	Banding Southern Blot Array‐CGH (here)	Denmark	McCormick et al. (1989) Petersen et al. (1990)	#045	#046
3	Proband	Non‐DS	F	1 yr	47,XX,+der(21),t(9;21)(34.1;q22.1)mat	Banding FISH Array‐CGH (here)	Italy	Mattina et al. (1997)	#076	#077
4	B.B.	DS	M	6 yrs	t(15;21)	Banding Array‐CHG	Alabama (US)	Finley et al. (1965), Hamm et al. (2015)	—	#002
5	Proband	DS	M	5 yrs	46,XY, idic(21)(q22.3)	FISH	India	Sheth et al. (2007)	—	#105
6	Patient	DS	F	2 yrs	46,XX, psu idic(21) (q22.3)	Banding FISH	Japan	Egashira et al. (2008)	—	#108
7	Proband	DS	M	2 mos	46,XY,der20t(20,21) (21qter‐>21q22::20p13‐>21qter)	Banding FISH Array‐CGH	Poland	Biaduń‐Popławska et al. (2014)	—	#127
8	Patient	Non‐DS	F	5 yrs	46,XX, dup21q22.11(chr21:32,583,901‐35,355,969;hg19)	Array‐CGH	Colorado (US)	Weisfeld‐Adams et al. (2016)	—	#130
9	Case 1	Non‐DS	F	2 yrs	46,XX, dup(21) q22.2q22.3	Banding Array‐CGH	Taiwan	Su et al. (2016)	—	#131
10	Case 2	Non‐DS	F	14 mos	46, XX, dup(21)q11.2q21	Banding Array‐CGH	Taiwan	Su et al. (2016)	—	#132

Array–based comparative genomic hybridization (array‐CGH) analysis was performed on DNA from three patients by using two different Agilent Technologies platforms (Agilent Technologies, Santa Clara, CA), following the manufacturer's protocol: SurePrint G3 Unrestricted CGH ISCA v2, with an average resolution of 60 kb, for MP01 and MP03 (cases 1 and 2, Table 1), and Human Genome CGH 60k Oligo Microarray Kit, with a median spatial resolution of 41.5 Kb, for proband case number 3 (Table 1). A graphical visualization of the results was provided by the Genomic Workbench software v.7.0 for the three patients and aberrations were called by the ADM1 algorithm with threshold at 6.0.

### 2.2 Bibliographic searches and case selection

We accurately searched for any reports of PT21 to integrate the new data in the previous PT21 map.

Bibliographic searches performed to build the starting map (Pelleri et al., 2016) were repeated in order to identify newly reported cases of PT21. "Mirror duplication chromosome 21" was used as additional PubMed query on the National Center for Biotechnology Information (NCBI) site (https://www.ncbi.nlm.nih.gov/pubmed/) and references cited in turn in the retrieved reports were examined. In addition, a search for "partial trisomy 21" through Google web search engine (https://www.google.com/) was performed.

Only new PT21 subjects with sufficient and unambiguous descriptions at the cytogenetic, molecular, and clinical levels were included in the study, following inclusion and exclusion criteria as previously described in detail (Pelleri et al., 2016). Briefly, the main cytogenetic inclusion criterion was the presence of a 21q partial duplication, excluding from the analysis cases presenting translocations and ring Hsa21 with a complete 21q, tetrasomies of Hsa21, mosaic trisomy 21, chromosomal rearrangements involving the X chromosome, and chromosomal alterations described in leukemic cell clones. For the molecular analysis criteria, the condition was a detailed and unambiguous description of the duplicated segment boundaries. At the clinical level, subjects were classified as "DS" or "non‐DS" according to the following criteria: (a) explicit statements found in the study; (b) whether authors judged recognizable DS as present or absent, irrespectively of other symptoms or signs associated to possibly concurrent aneuploidies of non‐Hsa21 chromosomal segments; (c) assessment of detailed phenotype description when present in the article.

The cases thus retrieved were numbered starting from 4 to 10 following chronological order of first description in literature.

### 2.3 Case descriptions

Main clinical data of the reanalyzed or reviewed cases are summarized in Table 1.

Briefly, the first two reanalyzed cases were subjects MP01 and MP03 (cases 1 and 2, Table 1). They were firstly reported in 1989 (McCormick et al., 1989) and 1990 (Petersen et al., 1990). According to the checklist of Jackson et al. (Jackson, North, & Thomas, 1976), a phenotypic score consistent with the clinical diagnosis of DS was reported for patient MP01, while patient MP03's phenotypic score is below the threshold for the clinical diagnosis of DS (Petersen et al., 1990).

The third reanalyzed case (case 3 in Table 1) was first reported in 1997 (Mattina et al., 1997) and we present clinical data from the follow‐up of the patient here. The proband is the only child of nonconsanguineous parents, born at the 40th week of gestation with eutocic delivery after an uncomplicated pregnancy; low‐risk triple test; birth weight was 3,020 g. The child was admitted to hospital for the first time at birth due to neonatal asphyxia and dysmorphic signs. The traditional karyotype through fluorescence in situ hybridization (FISH) was: 47, XX, +der21, t(9;21), but the patient was recognized as affected by a chromosomal syndrome due to a double partial trisomy 9q34.1‐>qter, 21pter‐>21q22.11. She was repeatedly admitted to the polyclinic due to frequent bronchopneumonic episodes and apnea crises. She is currently followed by annual follow‐up at the Medical Genetics Clinic. In agreement with the parents, clinical controls are carried out only within the limits of the "availability" of the girl. The girl shows a clinical picture with characteristics in agreement with partial trisomy 9q. Apparently, there are no clinical signs attributable to partial trisomy 21. Dysmorphic signs include brachycephaly, small anteverted ears with simple pavillon, straight forehead, divergent strabismus, proptosis, beak nose deviated to the left with narrow bridge, short philtrum, and "carp" mouth with thin lips. Prognathism, long and thin arms, hands and feet with arachnodactyly, and bilateral patella dislocation were also observed. Congenital malformations involved the heart (ventricular sept defect), brain (hypoplasia of the corpus callosum and subarachnoid space dilation), eyes (proptosis, nystagmus, divergent strabismus, pupil optic nerve pallor and hypoplasia, the patient underwent surgery to correct ectopia of crystalline lens and cataracts developed over years), skeleton (additional thoracic metameres with corresponding costal segments) and kidneys (hypoplasia‐dysplasia of the left kidney). During neonatal period and the first year of life, the patient did not thermoregulate and showed significant apnea crises, serious psychic and motor deficits, muscle hypotonia and hypotrophy, and afinalistic and uncoordinated hand movements. She pronounces "mom" and "dad", does not deambulate. Menarche was reported in September, 2009; irregular menstrual cycle. She presents with hand tremor.

Descriptions of seven cases (summary in Table 1, cases 4–10) retrieved by the new bibliographic search performed and mapped here have been reported in detail in the respective reports (Biaduń‐Popławska et al., 2014; Egashira et al., 2008; Finley, Finley, Rosecrans, & Phillips, 1965; Hamm, Carroll, Mikhail, Korf, & Finley, 2015; Sheth et al., 2007; Su et al., 2016; Weisfeld‐Adams, Tkachuk, Maclean, Meeks, & Scott, 2016).

### 2.4 Map framework building

We revised and updated all the sequence features of Hsa21 reported to build the starting map (Pelleri et al., 2016) (here Table S1): known Hsa21 genes, coordinates for single nucleotide polymorphisms (SNPs), sequence‐tagged sites, bacterial artificial chromosome clones, nucleotide positions determined by array‐CGH as limits of altered regions in individual subjects and cytogenetic band limits. All the genomic coordinates refer to the current Genome Reference Consortium (GRC) human genome assembly GRCh38, or hg38 (December 2013).

In summary, each spreadsheet row corresponds to a specific and relevant sequence feature on Hsa21 for a total of 724 sequence intervals (rows), providing anchor points useful to homogeneously map each cytogenetic feature described in the reports of PT21.

### 2.5 Comparative map building

For each subject studied, a column on the map file built as explained above was added, representing the structure of his/her Hsa21. Each row represents a specific sequence interval on Hsa21. Starting from the 125 PT21 cases previously described (Pelleri et al., 2016), we added refined limits of the duplicated portion for cases 1–3 to the map and mapped the additionally retrieved cases found with additional bibliographic searches performed here (Table 1, cases 4–10).

Each row represents a specific sequence interval on Hsa21 and for each subject with DS the corresponding cell was colored following this code: red = trisomic, therefore possible candidate as causing DS; green = disomic, therefore excluded as causing DS; blue = monosomic, considered as "not duplicated", therefore excluded as candidate; white = information not available with certainty. A complementary reasoning was used to color the cells representing sequence intervals when the subject presented cytogenetically with a segmental trisomy 21 in absence of a typical DS picture. In particular: red = disomic in non‐DS, therefore not excluded as causing DS; green = trisomic in non‐DS, therefore excluded as causing DS; blue = monosomic, considered as "not duplicated", therefore indirectly not excluded as candidate; white = information not available with certainty. Headings of non‐DS cases are highlighted in yellow.

### 2.6 Scoring system

A score was assigned to each interval sequence substantially following the scoring system applied by Lyle and coll. (Lyle et al., 2009), but attributing a lower score to not excluded regions in non‐DS subjects due to the fact that these regions would only be candidate regions indirectly.

A score of +1 was assigned to each trisomic (candidate) sequence interval in DS subjects, while +0.5 was assigned to disomic (not excluded) intervals in non‐DS subjects. A score of −1 was assigned to each sequence interval that was excluded as candidate for DS, being disomic in DS or trisomic in non‐DS subjects. Monosomic regions are considered as "not duplicated", therefore they should be excluded as candidates in DS subjects (score = −1) and indirectly not excluded in non‐DS (score = +0.5). For each sequence interval, the algebraic sum of the scores is calculated, generating the final score for the interval. The Excel macro and the Python scripts implementing the described algorithms for the calculations of the scores and for summarizing scores along Hsa21 regular intervals, respectively, are available upon request.

Higher scores indicate increased probability of association to DS. Detailed partial and final scores for each interval are reported in Table S1 at the right of the columns representing mapping for the 132 analyzed cases.

## 3 RESULTS

### 3.1 Molecular cytogenetic characterization

**Figure 1:**
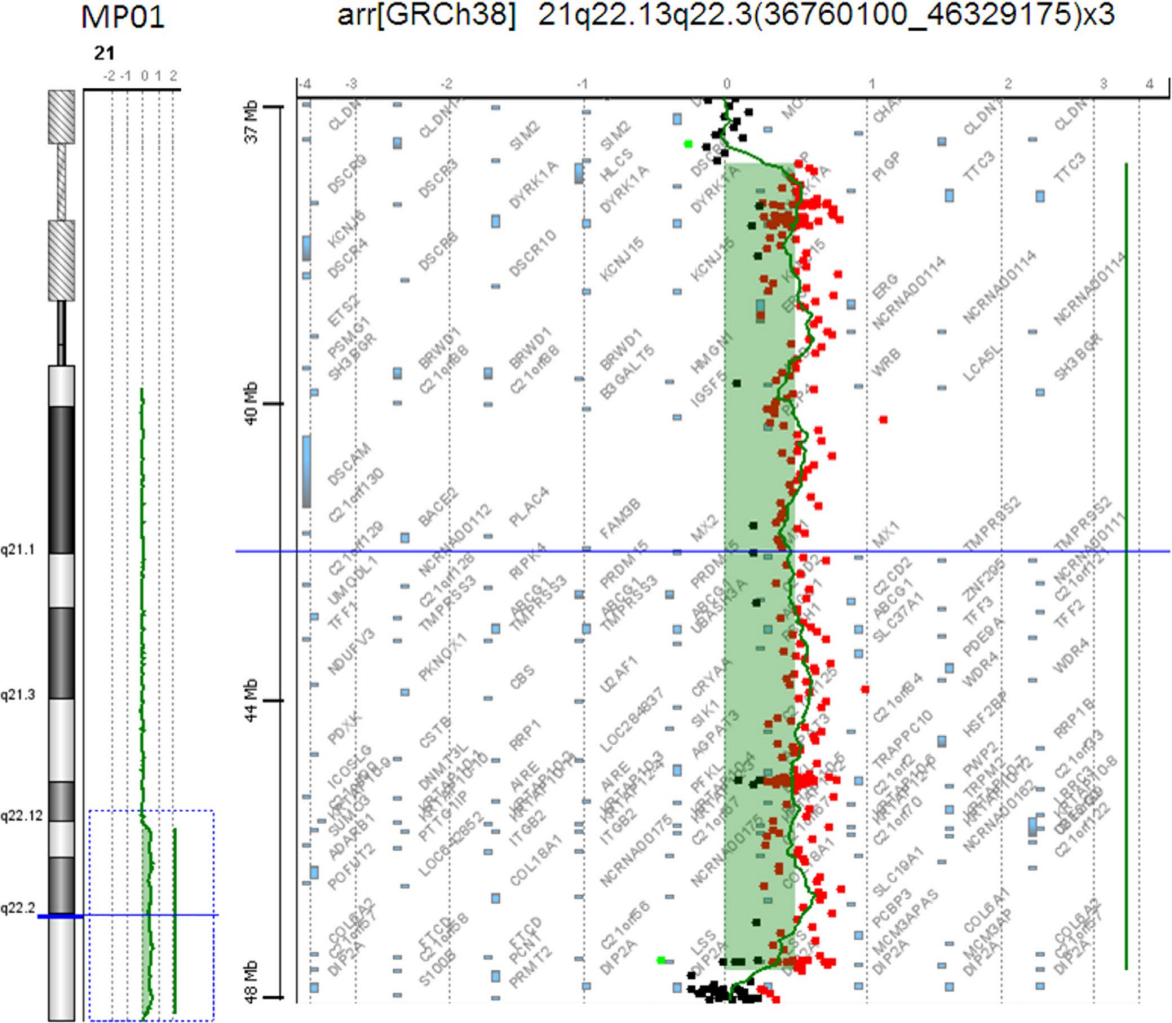
Array‐CGH analysis of DNA from patient MP01. Profile of chromosome 21 showing the duplication from 36,760,100 to 46,329,175 bases present in MP01 (reanalyzed case 1). Genomic coordinates refer to the current GRCh38 human assembly

The array‐CGH analysis of DNA from patient MP01 (case 1, Map ID here #044) revealed a duplication of Hsa21 from 36,760,100 to 46,329,175 bp (GRCh38) (Figure 1).

**Figure 2:**
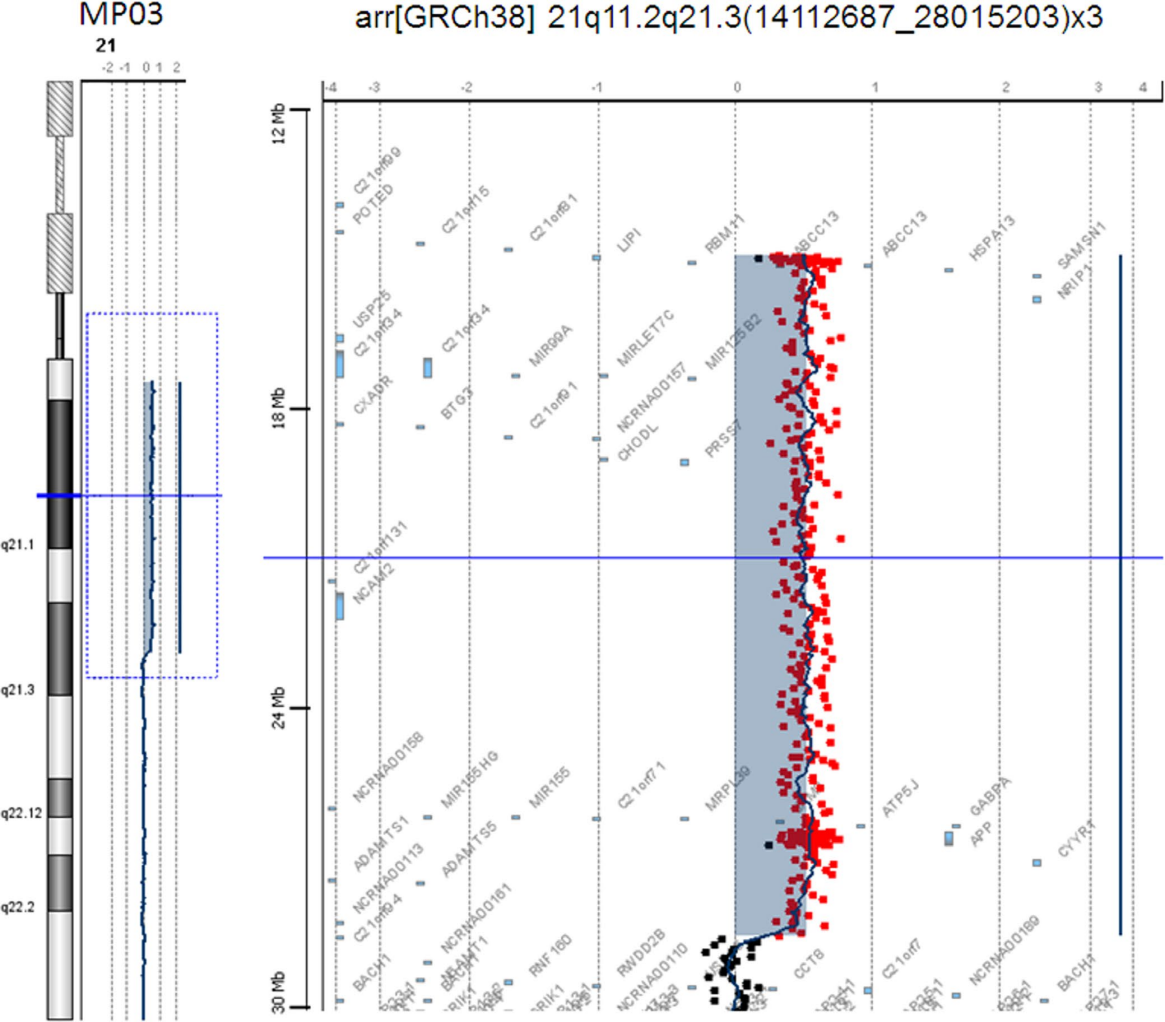
Array‐CGH analysis of DNA from patient MP03. Profile of chromosome 21 showing the duplication from 14,112,687 to 28,015,203 bases present in MP03 (reanalyzed case 2). Genomic coordinates refer to the current GRCh38 human assembly

The array‐CGH analysis of DNA from patient MP03 (case 2, Map ID here #046) revealed trisomy of Hsa21 from 14,112,687 to 28,015,203 bp (GRCh38) (Figure 2). The array did not allow evaluation of the Hsa21 portion upstream the proximal limit.

**Figure 3:**
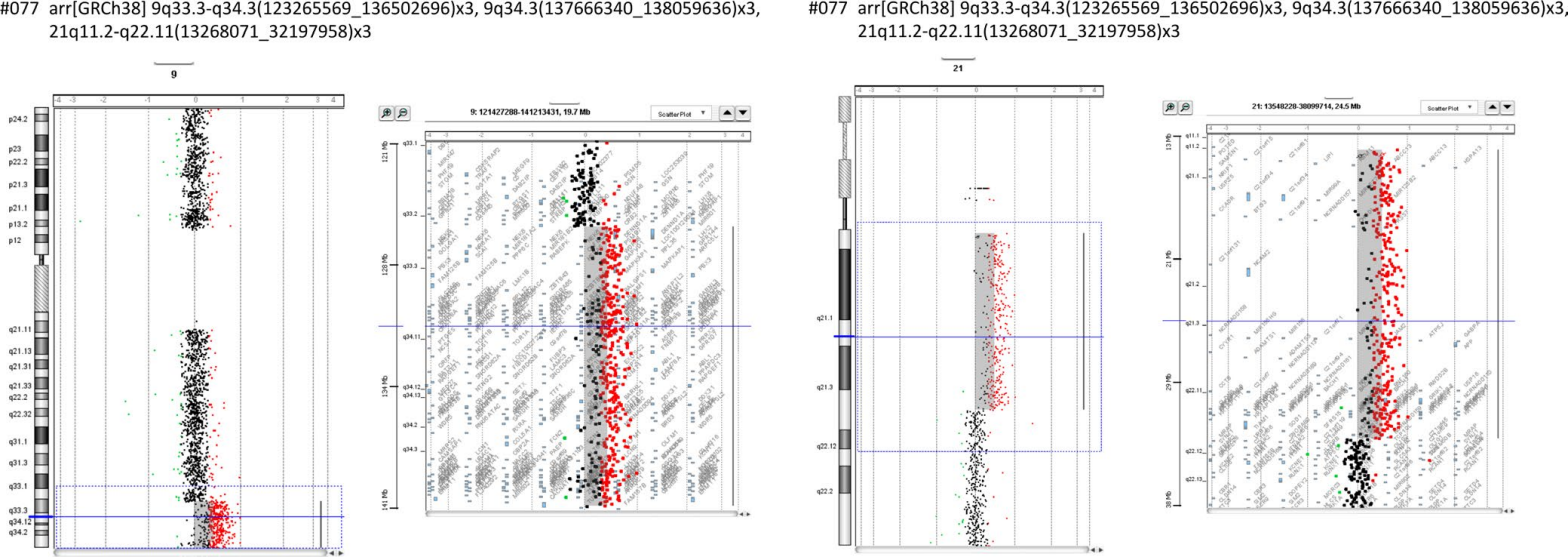
Array‐CGH analysis of DNA from patient #077. Profile of chromosomes 9 and 21 showing the duplication of chromosome 9 from 123,265,569 to 136,502,696 and from 137,666,340 to 138,059,636 bases and the duplication of chromosome 21 from 13,268,071 to 32,197,958 present in case #077 (reanalyzed case 3). Genomic coordinates refer to the current GRCh38 human assembly

Regarding case 3 (Map ID here #077), duplications of about 13.4 megabases (Mb) in the 9q33.3‐q34.3 chromosome region, of about 0.45 Mb in the 9q34.3 chromosome region, and of about 19 Mb in the 21q11.2‐q22.11 chromosomal region have been highlighted. According to ISCN 2016 nomenclature, the alterations may be described as: arr[GRCh38] 9q33.3‐q34.3(123265569_136502696)x3, 9q34.3(137666340_138059636)x3, 21q11.2‐q22.11(13268071_32197958)x3 (Figure 3).

These new limits have been recorded in the Table S1.

### 3.2 Bibliographic searches

Bibliographic searches performed to build the starting map (Pelleri et al., 2016) were repeated in order to identify newly reported cases of PT21, resulting in four newly described PT21 cases to be included (Table 1, cases 4 and 8–10) (Finley et al., 1965; Hamm et al., 2015; Su et al., 2016; Weisfeld‐Adams et al., 2016).

Using "mirror duplication chromosome 21" as additional PubMed query (Pelleri et al., 2016) and examining references cited in turn in the retrieved reports led to the retrieval of two additional PT21 cases (Egashira et al., 2008; Sheth et al., 2007) to be included here according to selection criteria detailed above (Table 1, cases 5 and 6).

In addition, a search for "partial trisomy 21" through Google web search engine revealed a new described PT21 report case (Biaduń‐Popławska et al., 2014) not included in PubMed to be included according to matching selection criteria (Table 1, case 7) detailed above.

In total, seven cases were added to the map (Table S1) following the new bibliographic search performed and inclusion/exclusion criteria described in Materials and Methods section. A complete list of all retrieved included and excluded reports of PT21 cases is available in the Supplementary References file. The complete description of all these PT21 cases is available in the Table S2, along with reasons for inclusion and exclusion.

### 3.3 PT21 comparative map building

The updated integrated comparative map obtained as described in the Materials and Methods section showing the localization of segmental anomalies of Hsa21 includes a total of 132 PT21 DS or non‐DS cases.

**Figure 4:**
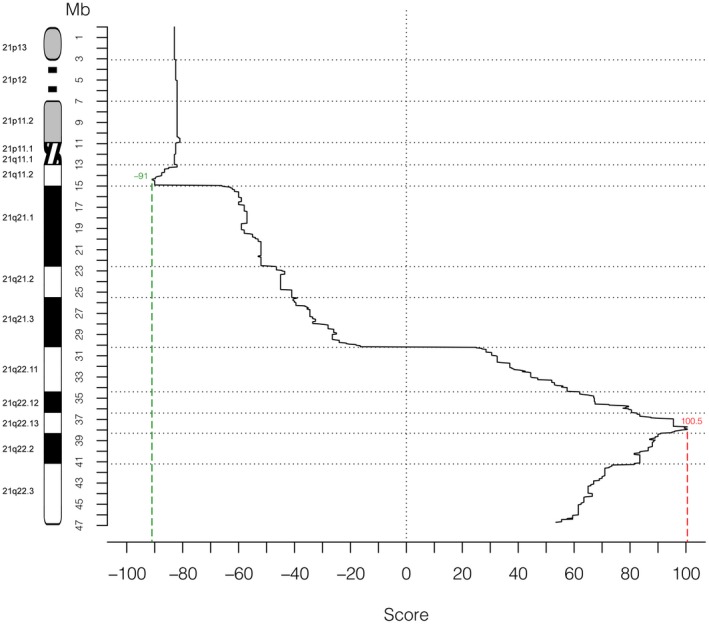
Genotype–phenotype correlation in 132 cases of partial trisomy 21. The X‐axis displays the score for association with DS for each sequence interval of 50 kb, shown as median of the values assigned to each map row (Table S1) that is comprised in each interval. The Y‐axis represents the position along Hsa21 (scale in megabases, Mb)

Scores for association with DS for each sequence interval are graphed in Figure 4. Complete data for each distinct sequence interval placed in the map are given in Table S1.

Analysis of the PT21 comparative map confirmed the exclusion of several regions of Hsa21 as associated to DS, in particular 21p, 21q11, and 21q21. Highest scores were found in the 21q22.13 and 21q22.2 sub‐bands (Figure 4). The 34‐kb interval from 37,929,229 to 37,963,130 previously described as the minimal region associated to DS and located on distal 21q22.13 (Pelleri et al., 2016), still has the highest scores, even when considering pure positively candidate scores not integrated with penalization/exclusion scores (Table S1). In addition, the duplication of this region is shared by all 92 DS subjects with available data about it and is absent in all 40 non‐DS subjects with available data about it, thus confirming consistency with the HR‐DSCR.

**Figure 5:**
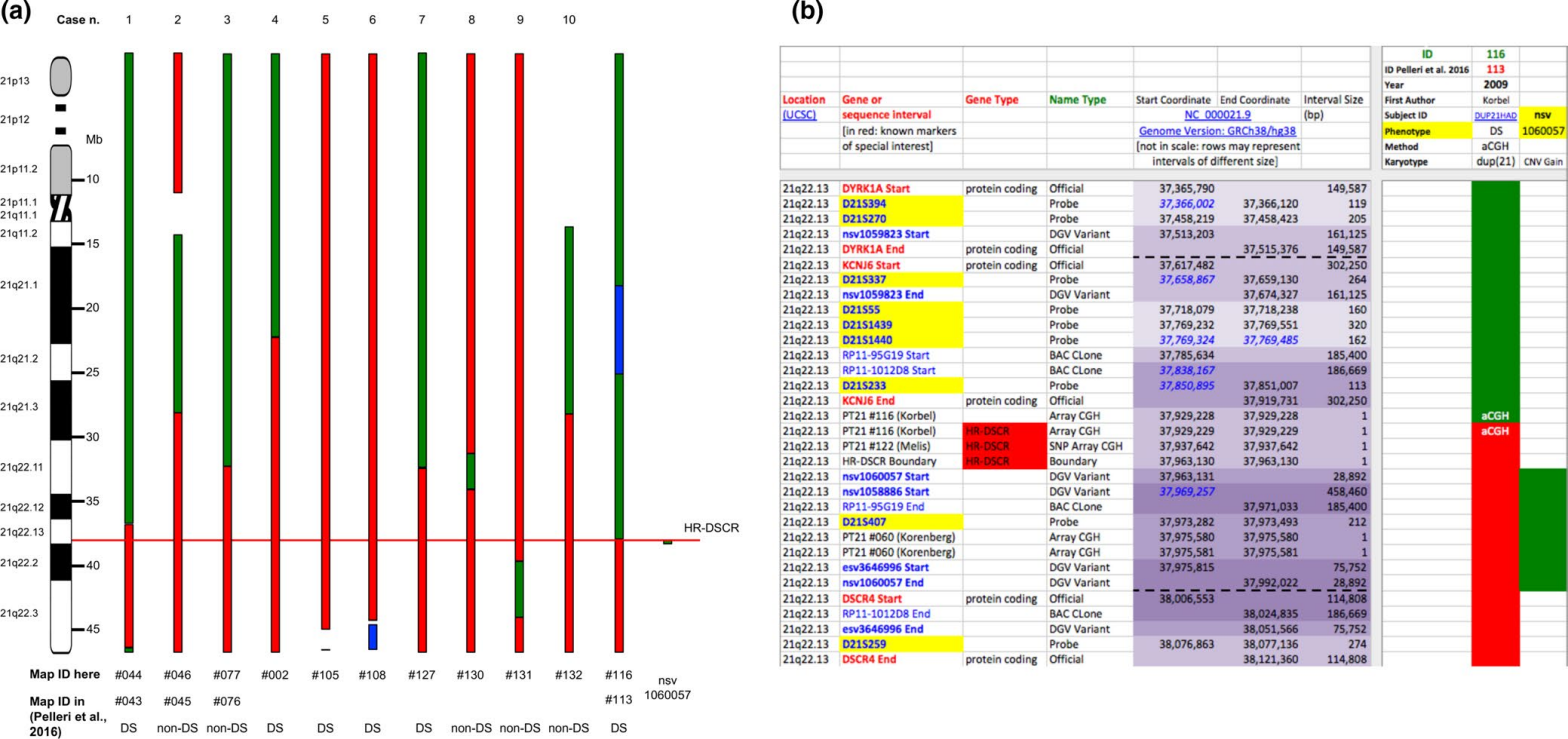
Concise outlook (a) of a portion of the partial trisomy 21 map. Each column represents the chromosome 21 structure (in megabases, Mb) of partial trisomy 21 subjects with or without Down syndrome (DS or non‐DS). For each subject the case number (n.), the updated map identifier (ID) and the corresponding previous map ID if present (Pelleri et al., 2016) are reported. The ten cases reanalyzed or reviewed here are reported together with Map ID #116 and the duplication coded as structural variant nsv1060057 which delimited the proximal and distal highly restricted DS critical region (HR‐DSCR, red continuous line) boundaries, respectively. Red boxes, candidate or not excluded regions; green boxes, excluded regions; white boxes: information not available with certainty; blue boxes, monosomic regions. A zoom‐in (b) of the case (#116 here and #113 in the previous study (Pelleri et al., 2016)) and the CNV (nsv1060057) strictly defining HR‐DSCR limits are shown here. Rows: Hsa21 sequence intervals (only those centered on HR‐DSCR are represented here). Red = candidate or not excluded regions; green = excluded regions. Levels of overlapping among intervals are indicated by increasingly darker violet color of the coordinates; blue italics indicate coordinates overlapping (Start or End) or nesting (Start and End) with the immediately previous interval (row). HR‐DSCR coordinates: 37,929,229–37,963,130. Complete map is available as Table S1

A concise, sample outlook of a portion of the map is depicted in Figure 5a, showing in particular the three reanalyzed cases (Table 1, cases 1–3; #044, #046, #077 Map ID here), two additionally retrieved cases (Table 1, cases 5 and 6; #105 and #108 Map ID here), and the five newly described cases (Table 1, cases 4 and 7–10; #002, #127, #130, #131, #132 Map ID here).

## 4 DISCUSSION

Over time, the concept of a "Down syndrome critical region" on Hsa21 has assumed different meanings. It was mainly criticized observing that a certain single duplicated region is not responsible for all or most DS features, that is, it is not sufficient to cause the full DS phenotype (Korbel et al., 2009; Lyle et al., 2009; Papoulidis et al., 2014). From this point of view, modeling the genotype‐phenotype correlations using PT21 cases has turned into the study of distinct phenotypic features, in order to associate each of them to a specific region most constantly duplicated in subjects presenting with the feature (Korbel et al., 2009; Korenberg et al., 1994; Lyle et al., 2009). This approach has certainly been successful in suggesting that specific Hsa21 subregions of various sizes are mostly associated to hypotonia (Lyle et al., 2009), to acute megakaryoblastic leukemia and transient myeloproliferative disorder (Korbel et al., 2009; Pelleri et al., 2014), and to CHD (Pelleri et al., 2017), among others. Due to high frequency of an Alzheimer‐like disease in DS and the location of the *APP* (amyloid beta precursor protein) gene on Hsa21, this phenotype represents a particularly suggestive example of the usefulness of PT21 study in defining genetic determinants for a phenotypic feature. Recently, it has been possible to confirm the obligatory role of *APP* in the clinical, biochemical, and neuropathological findings of Alzheimer‐like disease studying a case of PT21 with DS and without Alzheimer disease, lacking the *APP* duplication (Doran et al., 2017).

In addition to the search for genotype‐phenotype correlations aimed at dissecting single phenotypes, a few features regularly present at the highest frequency in subjects with DS can be considered so that the DSCR is viewed as the region which "suffices to induce the main phenotypic symptoms of the classic syndrome of trisomy 21" (Rethore, 1981). Therefore, using the diagnosis of DS itself as the phenotype to be mapped, the PT21 approach should point to the "minimal" Hsa21 region associated to the DS core features, notably ID. Actually, while any of the long list of symptoms and signs observed in DS may be absent in a proportion of the subjects, even at a high extent, ID and the typical facies are virtually universal, if cases with mosaicism are excluded. The genetic marker associated to these features should be, in principle, robust as the extra copy of the whole Hsa21. Dissection of small regions allowed by the variety of breakpoints delimiting duplicated regions in subjects with PT21 has repeatedly pointed to 21q22 band as associated to DS. Our systematic reanalysis of cytogenetic maps from 125 subjects with PT21, integrating them under a common and updated framework, has suggested that the duplication of a small 34‐kb region on distal 21q22.13 (HR‐DSCR) is fully coherent with the diagnosis of DS, while the disomic state of this region is consistent with a non‐DS phenotype (Pelleri et al., 2016).

In this work, we have tested if the HR‐DSCR limits could be refined by reanalyzing PT21 cases already included in the integrated map, and also if this model is prospectively consistent with additional PT21 cases not described at the moment of building the original map.

Regarding the reanalysis, we were successful in delineating duplicated regions from subjects MP01 (case 1, Map ID here #044), MP03 (case 2, Map ID here #046), and proband case number 3 (Map ID here #077), whose DNA was originally investigated by McCormick and coll. (McCormick et al., 1989), Petersen and coll. (Petersen et al., 1990), and Mattina and coll. (Mattina et al., 1997), respectively, and in this work has been subjected to array‐CGH. The array‐CGH analysis was able to clarify the breakpoints of the trisomic portions. The refinement of the limits in these reanalyzed cases was fully consistent with the previous reports and was fully consistent with the trisomic state of HR‐DSCR in the subject with DS and its disomic state in the subjects without a diagnosis of DS.

Regarding the verification of new cases, we accurately checked for any new reports published in the last two years and thus not considered in the original PT21 map. We further found five recent detailed descriptions (Table 1, cases 4 and 7–10) and two previously described cases not included in the original PT21 map (Table 1, cases 5 and 6) fulfilling criteria for the inclusion on the PT21 integrated map. Each of them deserved a specific publication due to the extreme rarity of each of these cases. The stringent criteria that we have defined for the establishment of genotype‐phenotype correlations in this type of study (Pelleri et al., 2016) have been very useful to guide the analysis of these new cases. Correlation of the clinical data to the cytogenetic map was consistent with the notion that all the subjects lacking the duplication of the HR‐DSCR were not diagnosed with DS, although these new cases did not allow further refinement of the region because the breakpoints of the duplicated portions were not within it. Remarkably, cases 1 and 9 (Figure 5a) alone would be sufficient to exclude very large portions of Hsa21 as associated to the diagnosis of DS delimiting a region of about 2.8 Mb (36,760,100–39,605,955) in which the HR‐DSCR is located in the center.

Interestingly, the girl with a duplication of 2.78 Mb of chromosome 21q22.11 (Table 1, case 8) (Weisfeld‐Adams et al., 2016) was considered borderline and without a clear diagnosis of DS. Regarding the two most constant features, the typical facies and the characteristic ID, the observation of the girl's picture (Figure 1 in (Weisfeld‐Adams et al., 2016): absence of oblique eyes and of gestalt recognition of the typical DS facies assessed through blind, independent evaluation by the clinicians involved in this work) and the phenotypic description lead to the non‐DS classification of the patient. In addition, the author reporting the case stated that, although showing some grade of developmental delay, the patient "lacked the happy, sociable affect observed in many children with DS". These findings are consistent with the lack of HR‐DSCR in this subject.

Taken together, all the findings presented here further support the concept that the distal part of the 21q22.13 sub‐band is strongly associated to diagnosis of DS while other regions are not, and they were all consistent with the HR‐DSCR model, although the limits of the reported duplications did not allow further refinement of the region or the confirmation of its narrowest extension. It should also be noted that variability both in Hsa21 (Sailani et al., 2013) or in other chromosomes (Priest et al., 2012) originating from CNVs or SNPs also contributes to both normal and DS phenotypes (Antonarakis, 2017).

A relevant problem remains to be the identification and characterization of the genetic determinants presumably located in the HR‐DSCR. Gene databases, as well as accurate transcriptome maps based on the use of known probes to obtain gene expression profiling data for organs involved in DS phenotypes such as hippocampus (Caracausi et al., 2016) or heart (Caracausi, Piovesan, Vitale, & Pelleri, 2017) might be of limited help in the hypothesis that the genetic determinants are presently uncharacterized. It is still possible that, due to the possible extremely low size of human introns (Piovesan et al., 2015), one or more spliced transcripts originate from this small region. RNA high‐throughput sequencing (Wang, Gerstein, & Snyder, 2009) or ENCODE (Encyclopedia of DNA Elements) project (ENCODE Project Consortium, 2012) data may offer the possibility to identify novel functional elements; however, we have not had to date success in using them to the aim of a better characterization of the HR‐DSCR. Other possibilities include the presence of unknown microRNA in the HR‐DSCR (Kozomara & Griffiths‐Jones, 2014), as the field of non‐coding RNA involved in DS pathogenesis has been recently explored (Karaca et al., 2017; Xu et al., 2013; Zhao, Jaber, Percy, & Lukiw, 2017) revealing the functions of several microRNA located on other Hsa21 regions with the capability to potentially regulate over 3,600 protein‐encoding genes (Alexandrov, Percy, & Lukiw, 2017; Li et al., 2012; Quinones‐Lombrana & Blanco, 2015).

While this work was in revision, an update in the Ensembl and UCSC genome browsers actually mapped two small new exons on the *KCNJ6* locus (OMIM # 600877) encoding potassium voltage‐gated channel subfamily J member 6, based on automated parsing of high throughput RNA sequencing (RNA‐Seq) data. Remarkably, this predicts a size extension for the *KCNJ6* locus from 292,216 bp as currently reported in NCBI Gene database (coordinates 37,624,223–37,916,438 in human genome map GRCh38.p12) to 497,787 bp (37,623,559–38,121,345). This transcript allows the prediction of a new intron which totally encompasses HR‐DSCR and preliminary experimental data confirm the existence of this intron. Interestingly, according to RNA‐Seq Expression Data from GTEx as reported in the UCSC browser, this transcript appears to be expressed at a high level in the brain and in the pituitary gland, in particular in the cerebellum, hippocampus, and cortex within the brain, regions well known for their specific alteration in DS (Stagni, Giacomini, Emili, Guidi, & Bartesaghi, 2018). While this update suggests the presence of transcribed DNA in the HR‐DSCR for the first time, further work will be necessary to characterize structure and function of this complex locus.

Moreover, the HR‐DSCR might function as a long‐range interactor with other chromosomes, although preliminary analysis of databases derived from high‐throughput chromosome conformation capture data (Hi‐C) (Durand et al., 2016) was not able to retrieve useful results (data not shown). An interesting possibility is to generate trisomy 21 cells with the selective deletion of a single copy of the HR‐DSCR to compare the features of fully trisomic, HR‐DSCR deleted and normal cells to prove that the critical region is exerting some function, for example, modifying the metabolic profile that has recently been found to be specifically altered in DS by metabolome analysis (Caracausi et al., 2018). The description of the CRISPR/Cas9 method (Bauer, Canver, & Orkin, 2015) may make the realization of such a sophisticated study model realistic (Harrison, Sanz, & Hollywood, 2016).

Therefore, while a relevant body of cytogenetic data obtained from the analysis of PT21 cases strongly suggests that there should be some critical elements for the pathogenesis of DS present in the distal part of the 21q22.13 sub‐band, further studies are needed for additional verification of the limits of the region in any case of PT21 reported in the future as well as for the characterization of functional loci within it. The success of these objectives might be critical to build a rational foundation for a treatment of DS ID.

In conclusion, the findings presented here represent the most comprehensive study on PT21 and further support the association of the HR‐DSCR with the diagnosis of DS, representing an unbiased validation of the original model that could be performed also in the future. Further studies are needed to identify and characterize genetic determinants presumably located in the HR‐DSCR and functionally associated to the critical manifestations of DS.

## CONFLICT OF INTEREST

The authors declare that they have no competing interests.

## AUTHORS' CONTRIBUTIONS

MCP designed the study. MCP, EC, and FA collected the data, built the integrated map, and performed the analysis. MBP, LT, and TM obtained and interpreted clinical and molecular data for the three cases reanalyzed here. PM performed the array‐CGH analysis for two cases and interpreted the data. GC and CL performed case selection and discussed the clinical data. MS analyzed and discussed the cytogenetic data. LV and MC performed the bibliographic data search and contributed to mapping data collection and analysis. AP developed the scoring system, the pertinent software and graphed the data. MCP, AP, and PS wrote the manuscript draft. AP and GC supervised the project. All authors contributed to the discussion of the data and to the Discussion section and read and approved the final manuscript.

## ETHICS APPROVAL

The patients described here were studied in the context of diagnostic genetic testing at the relative Institution as described in the original report for each subject.
