# Supplementary material for: Partial trisomy 21 map: Ten cases further supporting the highly restricted Down syndrome critical region (HR‐DSCR) on human chromosome 21
Source: Mol Genet Genomic Med. 2019 Jun 25;7(8):e797. doi: 10.1002/mgg3.797 (PMC6687668; doi:10.1002/mgg3.797)
Supplement: Supplementary file 2 [file MGG3-7-e797-s002.docx]

**Supplementary References**

**Table of contents**

**1. References for all cases with partial trisomy 21 described in Supplementary Material Table S2 and included in the integrated map (Supplementary Material Table S1)**

**2. References for all cases with partial trisomy 21 described in Supplementary Material Table S2 and excluded from the integrated map (Supplementary Material Table S1)**

**3. References for all cases with partial trisomy 21 excluded from the study due to mosaicism**

**4. References for all cases with partial trisomy 21 excluded from the study due to t(X;21) translocation**

**1. References for all cases with partial trisomy 21 described in Supplementary Material Table S2 and included in the integrated map (Supplementary Material Table S1)**

1. Abeliovich, D., Dagan, J., Lerer, I., Silberstein, S., Katznelson, M.B., Frydman, M. (1996) t(15;21)(q15;q22.1) pat resulting in partial trisomy and partial monosomy of chromosomes 15 and 21 in two offspring. Am. J. Med. Genet., 66, 45-51.
2. Aguinaga, M., Razo, G., Castro, J., Mayen-Molina, D.G. (2006) Prenatal diagnosis of trisomy 21 without the Down syndrome phenotype. Prenat. Diagn., 26, 1168-1171.
3. Ahlbom, B.E., Wahlstrom, J., Saalman, R., Wadelius, C., Anneren, G. (2003) Severe psychomotor retardation in a boy with a supernumerary derivative chromosome resulting in partial trisomy 21 and partial trisomy 7p. Ann. Genet., 46, 29-35.
4. Aula, P., Leisti, J., von Koskull, H. (1973) Partial trisomy 21. Clin. Genet., 4, 241-251.
5. Aviv, H., Lieber, C., Yenamandra, A., Desposito, F. (1997) Familial transmission of a deletion of chromosome 21 derived from a translocation between chromosome 21 and an inverted chromosome 22. Am. J. Med. Genet., 70, 399-403.
6. Barlow, G.M., Chen, X.N., Shi, Z.Y., Lyons, G.E., Kurnit, D.M., Celle, L., Spinner, N.B., Zackai, E., Pettenati, M.J., Van Riper, A.J. et al. (2001) Down syndrome congenital heart disease: a narrowed region and a candidate gene. Genet. Med., 3, 91-101.
7. Barnicoat, A.J., Bonneau, J.L., Boyd, E., Docherty, Z., Fennell, S.J., Huret, J.L., King, M., Maltby, E.L., McManus, S., Pilz, D.T. et al. (1996) Down syndrome with partial duplication and del (21) syndrome: study protocol and call for collaboration. Study I: Clinical assessment. Clin. Genet., 49, 20-27.
8. Blouin, J.L., Aurias, A., Creau-Goldberg, N., Apiou, F., Alcaide-Loridan, C., Bruel, A., Prieur, M., Kraus, J., Delabar, J.M., Sinet, P.M. (1991) Cytogenetic and molecular analysis of a de novo tandem duplication of chromosome 21. Hum. Genet., 88, 167-174.
9. Borgaonkar, D.S., Bias, W.B., Chase, G.A., Sadasivan, G., Herr, H.M., Golomb, H.M., Bahr, G.F., Kunkel, L.M. (1973) Identification of a C6-G21 translocation chromosome by the Q-M and Giemsa banding techniques in a patient with Down's syndrome, with possible assignment of Gm locus. Clin. Genet., 4, 53-57.
10. Braddock, S.R., Henley, K.M., Potter, K.L., Nguyen, H.G., Huang, T.H. (2000) Tertiary trisomy due to a reciprocal translocation of chromosomes 5 and 21 in a four-generation family. Am. J. Med. Genet., 92, 311-317.
11. Bruni, L., Capolino, R., Tozzi, M.C., Colloridi, F., Smacchia, M.P. (1996) Down syndrome with unusual chromosome translocation: case report and review. Ann. Genet., 39, 240-242.
12. Cantu, J.M., Hernandez, A., Plascencia, L., Vaca, G., Moller, M., Rivera, H. (1980) Partial trisomy and monosomy 21 in an infant with an unusual de novo 21/21 translocation. Ann. Genet., 23, 183-186.
13. Capkova, P., Misovicova, N., Vrbicka, D. (2014) Partial trisomy and tetrasomy of chromosome 21 without Down Syndrome phenotype and short overview of genotype-phenotype correlation. A case report. Biomed. Pa.p Med. Fac. Uni.v Palacky Olomouc Czech. Repub., 158, 321-325.
14. Cervenka, J., Gorlin, R.J., Djavadi, G.R. (1977) Down syndrome due to partial trisomy 21q. Clin. Genet., 11, 119-121.
15. Chadefaux, B., Allard, D., Rethore, M.O., Raoul, O., Poissonnier, M., Gilgenkrantz, S., Cheruy, C., Jerome, H. (1984) Assignment of human phosphoribosylglycinamide synthetase locus to region 21q221. Hum. Genet., 66, 190-192.
16. Chen, L.S., Xue, D., Xi, Z.M., Liu, D.N., Zou, P.S., Ma, M., Xia, Y., Chen, X.H., Qiu, G.B., Cao, D.H. (2015) A very rare case of trisomy 4q32.3-4q35.2 and trisomy 21q11.2-21q22.11 in a patient with recombinant chromosomes 4 and 21. Gene, 563, 72-75.
17. Conte, R.A., Luke, S., Verma, R.S. (1995) Characterization of a ring chromosome 21 by FISH-technique. Clin. Genet., 48, 188-191.
18. Cossec, J.C., Lavaur, J., Berman, D.E., Rivals, I., Hoischen, A., Stora, S., Ripoll, C., Mircher, C., Grattau, Y., Olivomarin, J.C. et al. (2012) Trisomy for synaptojanin1 in Down syndrome is functionally linked to the enlargement of early endosomes. Hum. Mol. Genet., 21, 3156-3172.
19. Daniel, A. (1979) Normal phenotype and partial trisomy for the G positive region of chromosome 21. J. Med. Genet., 16, 227-229.
20. De la Chapelle, A., Koivisto, M., Schroder, J. (1973) Segregating reciprocal (4;21) (q21;q21) translocation with proposita trisomic for parts of 4q and 21. J. Med. Genet., 10, 384-389.
21. Delabar, J.M., Theophile, D., Rahmani, Z., Chettouh, Z., Blouin, J.L., Prieur, M., Noel, B., Sinet, P.M. (1993) Molecular mapping of twenty-four features of Down syndrome on chromosome 21. Eur. J. Hum. Genet., 1, 114-124.
22. Dominguez, M.G., Rivera, H., Vasquez, A.I., Hernandez-Zaragoza, G., Rivas, F. (2001) Interchange trisomy 21 by t(1;21)(p22;q22)mat. Genet. Couns., 12, 363-367.
23. Eggermann, T., Schonherr, N., Spengler, S., Jager, S., Denecke, B., Binder, G., Baudis, M. (2010) Identification of a 21q22 duplication in a Silver-Russell syndrome patient further narrows down the Down syndrome critical region. Am. J. Med. Genet. A, 152a: 356-359.
24. El-Ruby, M., Hemly, N.A., Zaki, M.S. (2007) Maternal balanced translocation (4;21) leading to an offspring with partial duplication of 4q and 21q without phenotypic manifestations of Down syndrome. Genet. Couns., 18, 217-226.
25. Emberger, J.M., Lloret, R., Rossi, D. (1980) [Partial trisomy 21 with 45 chromosomes due to translocation of two chromosomes 21 onto a chromosome 14 : 45,XX-14,-21,+t(14q21q21q) (author's transl)]. Ann. Genet., 23, 179-180.
26. Forster-Gibson, C.J., Davies, J., MacKenzie, J.J., Harrison, K. (2001) Cryptic duplication of 21q in an individual with a clinical diagnosis of Down syndrome. Clin. Genet., 59, 438-443.
27. Fritz, B., Van Oorschot, B., Latta, E., Rehder, H. (1996) [Possibilities for false-negative findings in trisomy 21 screening with FISH]. Z Geburtshilfe Neonatol., 200, 191-198.
28. Fryns, J.P., de Waepenaert, E., Van den Berghe, H. (1980) Unusual translocation in Down syndrome. Acta Paediatr. Belg., 33, 47-49.
29. Gijsbers, A.C., van Haeringen, A., Bosch, C.A., Hansson, K., Verschuren, M., Bakker, E., Breuning, M.H., Ruivenkamp, C.A. (2010) A subtle familial translocation t(3;21)(p26.3;q22.3): an apparently healthy boy with a 3p deletion and 21q duplication. Cytogenet. Genome Res., 128, 245-249.
30. Gul, D., Ogur, G., Sayli, B.S., Gokcay, E. (2003) Double trisomy (1q32-->qter and 21pter-->q22) in a newborn female resulting from a maternal t(1;21)(q32;q22). Genet. Couns., 14, 437-438.
31. Habedank, M., Rodewald, A. (1982) Moderate Down's syndrome in three siblings having partial trisomy 21q22.2 to qter and therefore no SOD-1 excess. Hum. Genet., 60, 74-77.
32. Hagemeijer, A., Smit, E.M. (1977) Partial trisomy 21. Further evidence that trisomy of band 21q22 is essential for Down's phenotype. Hum. Genet., 38, 15-23.
33. Ilgin Ruhi, H., Tukun, A., Karabulut, H., Bayazit, P., Bokesoy, I. (2001) A Down syndrome case with a karyotype of 46,XY,rec(21)dup(21q)inv(21)(p11q22) derived from paternal pericentric inversion of chromosome 21. Clin. Genet., 59, 368-370.
34. Kieran, M.W., Vekemans, M., Robb, L.J., Sinsky, A., Outerbridge, E.W., Der Kaloustian, V.M. (1992) Portohepatic shunt in a Down syndrome patient with an interchange trisomy 47,XY,-2,+der(2),+der(21)t(2;21)(p13;q22.1)mat. Am. J. Med. Genet., 44, 288-292.
35. Knight, L.A., Yong, M.H., Tan, M., Ng, I.S. (1996) Subtle translocation (18;21) confirmed by FISH in a patient with Down syndrome. Clin. Genet., 50, 430-432.
36. Kondo, Y., Mizuno, S., Ohara, K., Nakamura, T., Yamada, K., Yamamori, S., Hayakawa, C., Ishii, T., Yamada, Y., Wakamatsu, N. (2006) Two cases of partial trisomy 21 (pter-q22.1) without the major features of Down syndrome. Am. J. Med. Genet. A, 140, 227-232.
37. Korbel, J.O., Tirosh-Wagner, T., Urban, A.E., Chen, X.N., Kasowski, M., Dai, L., Grubert, F., Erdman, C., Gao, M.C., Lange, K. et al. (2009) The genetic architecture of Down syndrome phenotypes revealed by high-resolution analysis of human segmental trisomies. Proc. Natl. Acad. Sci. U. S. A., 106, 12031-12036.
38. Korenberg, J.R. (1990) Molecular mapping of the Down syndrome phenotype. Prog. Clin. Biol. Res., 360, 105-115.
39. Korenberg, J.R., Bradley, C., Disteche, C.M. (1992) Down syndrome: molecular mapping of the congenital heart disease and duodenal stenosis. Am. J. Hum. Genet., 50, 294-302.
40. Korenberg, J.R., Chen, X.N., Schipper, R., Sun, Z., Gonsky, R., Gerwehr, S., Carpenter, N., Daumer, C., Dignan, P., Disteche, C. et al. (1994) Down syndrome phenotypes: the consequences of chromosomal imbalance. Proc. Natl. Acad. Sci. U. S. A., 91, 4997-5001.
41. Kosaki, R., Kosaki, K., Matsushima, K., Mitsui, N., Matsumoto, N., Ohashi, H. (2005) Refining chromosomal region critical for Down syndrome-related heart defects with a case of cryptic 21q22.2 duplication. Congenit. Anom. (Kyoto), 45, 62-64.
42. Kubien, E., Kleczkowska, A. (1978) Familial translocation t(lp--;21q+) associated with Down's syndrome. Hum. Genet., 40, 341-344.
43. Lazzaro, S.J., Speevak, M.D., Farrell, S.A. (2001) Recombinant Down syndrome: a case report and literature review. Clin. Genet., 59, 128-130.
44. Lee, J., Stanley, J.R., Vaz, S.A., Mulvihill, J.J., Wilson, P., Hopcus-Niccum, D., Li, S. (2005) Down syndrome with pure partial trisomy 21q22 due to a paternal insertion (4;21) uncovered by uncultured amniotic fluid interphase FISH. Am. J. Med. Genet. A, 132a, 206-208.
45. Lejeune, J., Berger, R., Vidal, O.R., Rethore, M.O. (1965) [A case of G-G translocation in tandem]. Ann. Genet., 8, 60-62.
46. Leonard, C., Gautier, M., Sinet, P.M., Selva, J., Huret, J.L. (1986) Two Down syndrome patients with rec(21),dupq,inv(21)(p11;q2109) from a familial pericentric inversion. Ann. Genet., 29, 181-183.
47. Leschot, N.J., Slater, R.M., Joenje, H., Becker-Bloemkolk, M.J., de Nef, J.J. (1981) SOD-A and chromosome 21. Conflicting findings in a familial translocation (9p24;21q214). Hum. Genet., 57, 220-223.
48. Lyle, R., Bena, F., Gagos, S., Gehrig, C., Lopez, G., Schinzel, A., Lespinasse, J., Bottani, A., Dahoun, S., Taine, L. et al. (2009) Genotype-phenotype correlations in Down syndrome identified by array CGH in 30 cases of partial trisomy and partial monosomy chromosome 21. Eur. J. Hum. Genet., 17, 454-466.
49. Maciejewski, E., Vigneron, J., Lambert, L., Bonnet, C., Hascoet, J.M. (2012) Down syndrome with partial trisomy of chromosome 21 because of a de-novo unbalanced translocation t(13;21)(q10;q22). Clin. Dysmorphol., 21, 200-203.
50. Martinoli, E., Zuccotti, G.V., Pogliani, L., Volonte, M., Venturin, M., Fortina, P., Ertel, A., Redaelli, S., Riva, P., Dalpra, L. (2010) A tandem duplication of chromosome 21 in a newborn showing a phenotype inconsistent with Down syndrome. Am. J. Med. Genet. A, 152a, 1043-1045.
51. Mattei, J.F., Mattei, M.G., Ardissone, J.P., Charvet, J.P., Chiaramello, W., Giraud, F. (1979) [Partial trisomy 21]. Arch. Fr. Pediatr., 36, 404-412.
52. Mattei, J.F., Mattei, M.G., Baeteman, M.A., Giraud, F. (1981) Trisomy 21 for the region 21q223: identification by high-resolution R-banding patterns. Hum. Genet., 56, 409-411.
53. Mattina, T., Pierluigi, M., Mazzone, D., Scardilli, S., Perfumo, C., Mollica, F. (1997) Double partial trisomy 9q34.1-->qter and 21pter-->q22.11: FISH and clinical findings. J. Med. Genet., 34, 945-948.
54. McCormick, M.K., Schinzel, A., Petersen, M.B., Stetten, G., Driscoll, D.J., Cantu, E.S., Tranebjaerg, L., Mikkelsen, M., Watkins, P.C., Antonarakis, S.E. (1989) Molecular genetic approach to the characterization of the "Down syndrome region" of chromosome 21. Genomics, 5, 325-331.
55. Melis, D., Genesio, R., Cappuccio, G., MariaGinocchio, V., Casa, R.D., Menna, G., Buffardi, S., Poggi, V., Leszle, A., Imperati, F. et al. (2011) Mental retardation, congenital heart malformation, and myelodysplasia in a patient with a complex chromosomal rearrangement involving the critical region 21q22. Am. J. Med. Genet. A, 155a, 1697-1705.
56. Mikiel-Kostyra, K., Czerski, P., Bartosz, G., Sito, A., Leyka, W. (1980) [Significance of the type of chromosome aberrations and biochemical disorders for diagnosis of Down's syndrome and the phenotype of partial trisomy 21]. Pediatr. Pol., 55, 23-32.
57. Miyazaki, K., Yamanaka, T., Ogasawara, N. (1987) A boy with Down's syndrome having recombinant chromosome 21 but no SOD-1 excess. Clin. Genet., 32, 383-387.
58. Moreira, L.M., Riegel, M. (2000) Two sibs with duplication of 4q31-->qter due to 3:1 meiotic disjunction and mild phenotype. Genet. Couns., 11, 249-259.
59. Nadal, M., Mila, M., Pritchard, M., Mur, A., Pujals, J., Blouin, J.L., Antonarakis, S.E., Ballesta, F., Estivill, X. (1996) YAC and cosmid FISH mapping of an unbalanced chromosomal translocation causing partial trisomy 21 and Down syndrome. Hum. Genet., 98, 460-466.
60. Nadal, M., Moreno, S., Pritchard, M., Preciado, M.A., Estivill, X., Ramos-Arroyo, M.A. (1997) Down syndrome: characterisation of a case with partial trisomy of chromosome 21 owing to a paternal balanced translocation (15;21) (q26;q22.1) by FISH. J. Med. Genet., 34, 50-54.
61. Nadal, M., Vigo, C.G., Melaragno, M.I., Andrade, J.A., Alonso, L.G., Brunoni, D., Pritchard, M., Estivill, X. (2001) Clinical and cytogenetic characterisation of a patient with Down syndrome resulting from a 21q22.1-->qter duplication. J. Med. Genet., 38, 73-76.
62. O'Donnell, J.J., Hall, B.D., Conte, F.A., Romanowski, J.C., Epstein, C.J. (1975) Down's syndrome: localization of locus to distal portion of long arm of chromosome 21. Pediat. Res., 9, 315.
63. Ohira, M., Ichikawa, H., Suzuki, E., Iwaki, M., Suzuki, K., Saito-Ohara, F., Ikeuchi, T., Chumakov, I., Tanahashi, H., Tashiro, K. et al. (1996) A 1.6-Mb P1-based physical map of the Down syndrome region on chromosome 21. Genomics, 33, 65-74.
64. Oliveira, R., Doria, S., Madureira, C., Lima, V., Almeida, C., Pinho, M.J., Ramalho, C., Matoso, E., Barros, A., Carreira, I.M. et al. (2013) Inv21p12q22del21q22 and intellectual disability. Gene, 517, 120-124.
65. Pangalos, C., Theophile, D., Sinet, P.M., Marks, A., Stamboulieh-Abazis, D., Chettouh, Z., Prieur, M., Verellen, C., Rethore, M.O., Lejeune, J. et al. (1992) No significant effect of monosomy for distal 21q22.3 on the Down syndrome phenotype in "mirror" duplications of chromosome 21. Am. J. Hum. Genet., 51, 1240-1250.
66. Papas, T.S., Watson, D.K., Sacchi, N., Fujiwara, S., Seth, A.K., Fisher, R.J., Bhat, N.K., Mavrothalassitis, G., Koizumi, S., Jorcyk, C.L. et al. (1990) ETS family of genes in leukemia and Down syndrome. Am. J. Med. Genet. Suppl., 7, 251-261.
67. Park, J.P., Wurster-Hill, D.H., Andrews, P.A., Cooley, W.C., Graham, J.M. Jr. (1987) Free proximal trisomy 21 without the Down syndrome. Clin. Genet., 32, 342-348.
68. Pastva, M., Corwin, E.J., Morin, K. (2004) Down syndrome with an unusual etiology: case report and review. J. Am. Acad. Nurse Pract., 16, 244-250.
69. Pellissier, M.C., Laffage, M., Philip, N., Passage, E., Mattei, M.G., Mattei, J.F. (1988) Trisomy 21q223 and Down's phenotype correlation evidenced by in situ hybridization. Hum. Genet., 80, 277-281.
70. Petersen, M.B., Tranebjaerg, L., McCormick, M.K., Michelsen, N., Mikkelsen, M., Antonarakis, S.E. (1990) Clinical, cytogenetic, and molecular genetic characterization of two unrelated patients with different duplications of 21q. Am. J. Med. Genet. Suppl., 7, 104-109.
71. Pfeiffer, R.A., Kessel, E.K., Soer, K.H. (1977) Partial trisomies of chromosome 21 in man. Two new observations due to translocations 19;21 and 4;21. Clin. Genet., 11, 207-213.
72. Poissonnier, M., Saint-Paul, B., Dutrillaux, B., Chassaigne, M., Gruyer, P., de Blignieres-Strouk, G. (1976) [Partial trisomy 21 (21q21 - 21q22.2)]. Ann. Genet., 19, 69-73.
73. Prasher, V.P., Farrer, M.J., Kessling, A.M., Fisher, E.M., West, R.J., Barber, P.C., Butler, A.C. (1998) Molecular mapping of Alzheimer-type dementia in Down's syndrome. Ann. Neurol., 43, 380-383.
74. Pueschel, S.M., Padre-Mendoza, T., Ellenbogen, R. (1980) Partial trisomy 21. Clin. Genet., 18, 392-395.
75. Rahmani, Z., Blouin, J.L., Creau-Goldberg, N., Watkins, P.C., Mattei, J.F., Poissonnier, M., Prieur, M., Chettouh, Z., Nicole, A., Aurias, A. et al. (1989) Critical role of the D21S55 region on chromosome 21 in the pathogenesis of Down syndrome. Proc. Natl. Acad. Sci. U. S. A., 86, 5958-5962.
76. Rahmani, Z., Blouin, J.L., Creau-Goldberg, N., Watkins, P.C., Mattei, J.F., Poissonnier, M., Prieur, M., Chettouh, Z., Nicole, A., Aurias, A. et al. (1990) Down syndrome critical region around D21S55 on proximal 21q22.3. Am. J. Med. Genet. Suppl., 7, 98-103.
77. Raoul, O., Carpentier, S., Dutrillaux, B., Mallet, R., Lejeune, J. (1976) [Partial trisomy of chromosome 21 by maternal translocation t(15;21) (q26.2; q21)]. Ann. Genet., 19, 187-190.
78. Rethore, M.O., Lafourcade, J., Couturier, J., Harpey, J.P., Hamet, M., Engler, R., Alcindor, L.G., Lejeune, J. (1982) [Increased activity of adenine phosphoribosyl transferase in a child trisomic for 16q22.2 to 16qter due to malsegregation of a t(16;21) (q22.2;q22;2)pat]. Sem. Hop., 58, 2639-2645.
79. Ronan, A., Fagan, K., Christie, L., Conroy, J., Nowak, N.J., Turner, G. (2007) Familial 4.3 Mb duplication of 21q22 sheds new light on the Down syndrome critical region. J. Med. Genet., 44, 448-451.
80. Rovelet-Lecrux, A., Hannequin, D., Raux, G., Le Meur, N., Laquerriere, A., Vital, A., Dumanchin, C., Feuillette, S., Brice, A., Vercelletto, M. et al. (2006) APP locus duplication causes autosomal dominant early-onset Alzheimer disease with cerebral amyloid angiopathy. Nat. Genet., 38, 24-26.
81. Sanchez, O., Mamunes, P., Yunis, J.J. (1977) Partial trisomy 20 (20q13) and partial trisomy 21 (21pter leads to 21q21.3). J. Med. Genet., 14, 459-462.
82. Sato, D., Kawara, H., Shimokawa, O., Harada, N., Tonoki, H., Takahashi, N., Imai, Y., Kimura, H., Matsumoto, N., Ariga, T. et al. (2008) A girl with Down syndrome and partial trisomy for 21pter-q22.13: a clue to narrow the Down syndrome critical region. Am. J. Med. Genet. A, 146a, 124-127.
83. Scott, J.A., Wenger, S.L., Steele, M.W., Chakravarti, A. (1995) Down syndrome consequent to a cryptic maternal 12p;21q chromosome translocation. Am. J. Med. Genet., 56, 67-71.
84. Sebastio, G., Perone, L., Guzzetta, V., Sebastio, L., Vicari, L., Della Casa, R., Gurrieri, F., Zappata, S., Pomponi, M.G., Mazzei, A. et al. (1996) Molecular and cytogenetic characterization of a recurrent unbalanced translocation (4;21)(p16.3;q22.1): relevance to the Wolf-Hirschhorn and Down syndrome critical regions. Am. J. Med. Genet., 63, 366-372.
85. Shabtai, F.S., Schwartz, A., Klar, D., Hart, J., Dar, H., Kessler, E., Halbrecht, I. (1990) Free proximal trisomy 21 in the mother and malformation syndrome in the son. Am. J. Med. Genet. Suppl., 7, 182-185.
86. Sinet, P.M., Couturier, J., Dutrillaux, B., Poissonnier, M., Raoul, O., Rethore, M.O., Allard, D., Lejeune, J., Jerome, H. (1976) [Trisomy 21 and superoxide dismutase-1 (IPO-A). Tentative localization of sub-band 21Q22.1]. Exp. Cell. Res., 97, 47-55.
87. Sinet, P.M., Theophile, D., Rahmani, Z., Chettouh, Z., Blouin, J.L., Prieur, M., Noel, B., Delabar, J.M. (1994) Mapping of the Down syndrome phenotype on chromosome 21 at the molecular level. Biomed. Pharmacother., 48, 247-252.
88. Strah, D., Veble, A., Rudolf, G., Writzl, K., Gersak, K. (2008) A Down syndrome female infant with partial trisomy of chromosome 21--abnormal nuchal translucency screening test. Genet. Couns., 19, 429-432.
89. Takahashi, T., Inoue, A., Yoshimoto, J., Kanamitsu, K., Taki, T., Imada, M., Yamada, M., Ninomiya, S., Toki, T., Terui, K. et al. (2015) Transient myeloproliferative disorder with partial trisomy 21. Pediatr. Blood Cancer, 62, 2021-2024.
90. Tardy, E.P., Toth, A., Kosztolanyi, G. (1997) Prenatal exclusion of segmental trisomy in familial chromosome 21 pericentric inversion by fluorescence in situ hybridization. Prenat. Diagn., 17, 871-873.
91. Vaglio, A., Milunsky, A., Quadrelli, A., Huang, X.L., Maher, T., Mechoso, B., Martinez, S., Pagano, S., Bellini, S., Costabel, M. et al. (2010) Clinical, cytogenetic, and molecular characterization of a girl with some clinical features of Down syndrome resulting from a pure partial trisomy 21q22.11-qter due to a de novo intrachromosomal duplication. Genet. Test. Mol. Biomarkers, 14, 57-65.
92. Valero, R., Marfany, G., Gil-Benso, R., Ibanez, M.A., Lopez-Pajares, I., Prieto, F., Rullan, G., Sarret, E., Gonzalez-Duarte, R. (1999) Molecular characterisation of partial chromosome 21 aneuploidies by fluorescent PCR. J. Med. Genet., 36, 694-699.
93. Valetto, A., Bertini, V., Toschi, B., Simi, P. (2013) A 47,XX,+der(21)t(8;21)(q24.2;q21.1) karyotype in a patient with mild intellectual disability, cleft lip, Hashimoto thyroiditis and hirsutism. Am. J. Med. Genet. A, 161a, 2389-2392.
94. Villa, N., Bentivegna, A., Ertel, A., Redaelli, S., Colombo, C., Nacinovich, R., Broggi, F., Lissoni, S., Bungaro, S., Addya, S. et al. (2011) A de novo supernumerary genomic discontinuous ring chromosome 21 in a child with mild intellectual disability. Am. J. Med. Genet. A, 155a, 1425-1431.
95. Williams, C.A., Frias, J.L., McCormick, M.K., Antonarakis, S.E., Cantu, E.S. (1990) Clinical, cytogenetic, and molecular evaluation of a patient with partial trisomy 21 (21q11-q22) lacking the classical Down syndrome phenotype. Am. J. Med. Genet. Suppl, 7, 110-114.
96. Williams, J.D., Summitt, R.L., Martens, P.R., Kimbrell, R.A. (1975) Familial Down syndrome due to t(10;21) translocation: evidence that the Down phenotype is related to trisomy of a specific segment of chromosome 21. Am. J. Hum. Genet., 27, 478-485.
97. Weisfeld-Adams, J.D., Tkachuk, A.K., Maclean, K.N., Meeks, N.L., Scott, S.A. (2016) A de novo 2.78-Mb duplication on chromosome 21q22.11 implicates candidate genes in the partial trisomy 21 phenotype. NPJ. Genom. Med.
98. Su, M.T., Kuan, L.C., Chou, Y.Y., Tan, S.Y., Kuo, T.C., Kuo, P.L. (2016) Partial trisomy of chromosome 21 without the Down syndrome phenotype. Prenat. Diagn. 36, 492-495
99. Hamm, J.A., Carroll, A.J., Mikhail, F.M., Korf, B.R., Finley, W.H. (2015) Partial trisomy 21: a fifty-year follow-up visit. Am. J. Med. Genet. A. 167, 1610-1613
100. Finley, S.C., Finley, W.H., Rosecrans, C.J., Phillips, C. (1965) Exceptional intelligence in a mongoloid child of a family with a 13-15/partial 21 (D-partial G) translocation. N. Engl. J. Med. 272, 1089-1092.
101. Biaduń-Popławska, A., Jamsheer, A., Henkelman, M., Tuziak, M., Pietrzyk, A., Piotrowski, K.,Giżewska, M., Walczak , M. and Zajączek, S. (2014) Down Syndrome Phenotype in a Child with Partial Trisomy of Chromosome 21 and Paternally Derived Translocation t (20p; 21q). Gen Med (Los Angel) 2, 149.
102. Egashira, M., Kondoh, T., Kawara, H., Motomura, H., Tagawa, M., Harada, N., Moriuchi, H. (2008) Mirror duplication of chromosome 21 with complete phenotype of Down syndrome. Pediatr. Int. Aug. 50, 597-599.
103. Sheth, F.J., Radhakrishna, U., Morris, M.A, Blouin, J.L, Sheth, J.J, Multani, A., Antonarakis, E.S,(2007) Cytogenetic, Molecular and FISH Analysis of an Isodicentric Chromosome 21 idic(21)(q22.3) in a Mildly-Affected Patient with Down Syndrome. Int. J. Hum. Genet. 7, 215-218.

**2. References for all cases with partial trisomy 21 described in Supplementary Material Table S2 and excluded from the integrated map (Supplementary Material Table S1)**

1. Barbi, G., Kennerknecht, I., Wohr, G., Avramopoulos, D., Karadima, G., Petersen, M.B. (2000) Mirror-symmetric duplicated chromosome 21q with minor proximal deletion, and with neocentromere in a child without the classical Down syndrome phenotype. Am. J. Med. Genet., 91, 116-122.
2. Bartsch, O., Hinkel, G.K., Petersen, M.B., Konig, U., Bugge, M., Mikkelsen, M., Avramopoulos, D., Morris, M., Antonarakis, S.E. (1997) A large family with subtelomeric translocation t(18;21)(q23;q22.1) and molecular breakpoint in the Down syndrome critical region. Hum. Genet., 100, 669-675.
3. Cetin, Z., Yakut, S., Mihci, E., Manguoglu, A.E., Berker, S., Keser, I., Luleci, G. (2012) A patient with Down syndrome with a de novo derivative chromosome 21. Gene, 507, 159-164.
4. Crombez, E.A., Dipple, K.M., Schimmenti, L.A., Rao, N. (2005) Duplication of the Down syndrome critical region does not predict facial phenotype in a baby with a ring chromosome 21. Clin. Dysmorphol., 14, 183-187.
5. Delabar, J.M., Sinet, P.M., Chadefaux, B., Nicole, A., Gegonne, A., Stehelin, D., Fridlansky, F., Creau-Goldberg, N., Turleau, C., de Grouchy, J. (1987) Submicroscopic duplication of chromosome 21 and trisomy 21 phenotype (Down syndrome). Hum. Genet., 76, 225-229.
6. Fraisse, J., Philip, T., Bertheas, M.F., Lauras, B. (1986) Six cases of partial duplication-deficiency 21 syndrome: 21(dupq22delp23) due to maternal pericentric inversion: inv(21)(p12;q22). A family study. Ann. Genet., 29, 177-180.
7. Habedank, M., Kampe, G. (1975) Familial translocation t(3p-;21q+) associated with both Down's and Sturge-Weber's syndrome in unbalanced state. Humangenetik, 29, 207-216.
8. Horn, D., Neitzel, H., Tonnies, H., Kalscheuer, V., Kunze, J., Hinkel, G.K., Bartsch, O. (2003) Familial MCA/MR syndrome due to inherited submicroscopic translocation t(18;21)(q22.1q21.3) with breakpoint at the Down syndrome critical region. Am. J. Med. Genet. A, 117a, 236-244.
9. Huret, J.L., Delabar, J.M., Marlhens, F., Aurias, A., Nicole, A., Berthier, M., Tanzer, J., Sinet, P.M. (1987) Down syndrome with duplication of a region of chromosome 21 containing the CuZn superoxide dismutase gene without detectable karyotypic abnormality. Hum. Genet., 75, 251-257.
10. Matsubara, T., Nakagome, Y., Ogasawara, N., Oka, S., Yokochi, T. (1982) Maternally transmitted extra ring (21) chromosome in a boy with Down's syndrome. Hum. Genet., 60, 78-79.
11. Niebuhr, E. (1974) Down's syndrome. The possibility of a pathogenetic segment on chromosome no. 21. Humangenetik, 21, 99-101.
12. Papoulidis, I., Papageorgiou, E., Siomou, E., Oikonomidou, E., Thomaidis, L., Vetro, A., Zuffardi, O., Liehr, T., Manolakos, E., Vassilis, P. (2014) A patient with partial trisomy 21 and 7q deletion expresses mild Down syndrome phenotype. Gene, 536, 441-443.
13. Tlili, A., Hoischen, A., Ripoll, C., Benabou, E., Badel, A., Ronan, A., Touraine, R., Grattau, Y., Stora, S., van Bon, B. et al. (2012) BDNF and DYRK1A are variable and inversely correlated in lymphoblastoid cell lines from Down syndrome patients. Mol. Neurobiol., 46, 297-303.
14. Wahlstrom, J., Djerg, S. (1976) A familial tandem translocation (15;21) (q11;q22) in a case of Down's syndrome. J. Ment. Defic. Res., 20, 171-178.
15. Aleksiūnienė, B., Matulevičiūtė, R , Matulevičienė, A., Burnytė, B., Krasovskaja, N.,Ambrozaitytė, L., Mikštienė, V., Dirsė, V., Utkus, A., Kučinskas, V. (2017) Opposite chromosomeconstitutions due to a familial translocation t(1;21)(q43;q22) in 2 cousins with development delay and congenital anomalies: A case report. Medicine (Baltimore) 96, e6521.
16. Mekkawy, M.K., Mazen, I.M., Kamel, A.K., Vater, I., Zaki, M.S.(2016) Genotype/phenotype correlation in a female patient with 21q22.3 and 12p13.33 duplications. Am. J. Med. Genet. A. 170A, 1050-1058.
17. Ha, T.M., Nguyen, V.N., Lindor, N.M., Meyer, R.G., Rai, R., Velagaleti, G.V. (2010) A case of Down syndrome with mirror-image duplication of chromosome 21. Am. J. Med. Genet. A. 152A, 1580-1582.
18. Stoll, C., Pennerath, A., Lausecker C.(1982) A case of Down's syndrome resulting from mirror duplication of chromosome 21. Eur. J. Pediatr. 138, 80-81.

**3. References for all cases with partial trisomy 21 excluded from the study due to mosaicism**

1. Bertini, V., Valetto, A., Uccelli, A., Tarantino, E., Simi, P. (2008) Ring chromosome 21 and reproductive pattern: a familial case and review of the literature. Fertil. Steril., 90, 2004.e1-2004.e5.
2. Clarke, M.J., Thomson, D.A., Griffiths, M.J., Bissenden, J.G., Aukett, A., Watt, J.L. (1989) An unusual case of mosaic Down's syndrome involving two different Robertsonian translocations. J. Med. Genet., 26, 198-201.
3. Falik-Borenstein, T.C., Pribyl, T.M., Pulst, S.M., Van Dyke, D.L., Weiss, L., Chu, M.L., Kraus, J., Marshak, D., Korenberg, J.R. (1992) Stable ring chromosome 21: molecular and clinical definition of the lesion. Am. J. Med. Genet., 42, 22-28.
4. Fisher, A.M., Al-Gazali, L., Pramathan, T., Quaife, R., Cockwell, A.E., Barber, J.C., Earnshaw, W.C., Axelman, J., Migeon, B.R., Tyler-Smith, C. (1997) Centromeric inactivation in a dicentric human Y;21 translocation chromosome. Chromosoma, 106, 199-206.
5. Hongell, K., Airaksinen, E. (1972) A Gq deletion in a girl with Down's syndrome. Hum. Hered., 22, 80-85.
6. Howell, R.T., McDermott, A., Gardner, A., Dickinson, V. (1984) Down's syndrome with a recombinant tandem duplication of chromosome 21 derived from a maternal ring. J. Med. Genet., 21, 310-314.
7. Ieshima, A., Ogasawara, N., Yamamoto, Y., Kuroki, Y. (1980) A case of r(21) with stigmata of atypical Down syndrome. Hum. Genet., 55, 65-69.
8. Kazazian, H.H. Jr., Antonarakis, S.E., Wong, C., Trusko, S.P., Stetten, G., Oliver, M., Potter, M.J., Gusella, J.F., Watkins, P.C. (1985) Ring chromosome 21: characterization of DNA sequences at sites of breakage and reunion. Ann. N. Y. Acad. Sci., 450, 33-42.
9. Kennerknecht, I., Barbi, G., Vogel, W. (1990) Maternal transmission of ring chromosome 21. Hum. Genet., 86, 99-101.
10. Keppler-Noreuil, K.M., Welch, J.L., Major, H.J., Qiau, Q., Jordan, D.K., Patil, S.R. (2002) Atypical Down syndrome phenotype with severe developmental delay, hypertonia, and seizures in a child with translocation trisomy 21. Dev. Med. Child. Neurol., 44, 64-67.
11. Krasikov, N., Takaesu, N., Hassold, T., Knops, J.F., Finley, W.H., Scarbrough, P. (1992) Molecular and cytogenetic investigation of complex tissue-specific duplication and loss of chromosome 21 in a child with a monosomy 21 phenotype. Am. J. Med. Genet., 43, 554-560.
12. Matsumoto, N., Niikawa, N., Mikawa, M. (1995) Confirmation of Down syndrome critical region by FISH analysis in a patient with add(21) (p11). Am. J. Med. Genet., 59, 521-522.
13. McCreanor, H.R. (1979) Achievement by the genetically disabled: a history of social and medical aid to a patient with partial Down's syndrome. N. Z. Med. J., 90, 292-293.
14. Parloir, C., Fryns, J.P., Van den Berghe, H. (1979) Down's syndrome in brother and sister without evident trisomy 21. Hum. Genet., 51, 227-230.
15. Richer, C.L., Fitch, N., Sitahal, S., Murer-Orlando, M., Jean, P. (1981) Analysis of banding patterns in a case of ring chromosome 21. Am. J. Med. Genet., 10, 323-331.
16. Schmid, W., Tenconi, R., Baccichetti, C., Caufin, D., Schinzel, A. (1983) Ring chromosome 21 in phenotypically apparently normal persons: report of two families from Switzerland and Italy. Am. J. Med. Genet., 16, 323-329.
17. Stankiewicz, P., Bocian, E., Jakubow-Durska, K., Obersztyn, E., Lato, E., Starke, H., Mroczek, K., Mazurczak, T. (2000) Identification of supernumerary marker chromosomes derived from chromosomes 5, 6, 19, and 20 using FISH. J. Med. Genet., 37, 114-120.
18. Van Keuren, M.L., Stewart, G.D., Bradley, C.M., Kurnit, D.M., Neve, R.L., Watkins, P.C., Tanzi, R.E., Gusella, J.F., Patterson, D. (1989) Characterization of an unusual and complex chromosome 21 rearrangement using somatic cell genetics and cloned DNA probes. Am. J. Med. Genet., 33, 369-375.
19. Verma, R.S., Peakman, D.C., Robinson, A., Lubs, H.A. (1977) Two cases of Down syndrome with unusual de novo translocation. Clin. Genet., 11, 227-234.
20. Zhang, H.Z., Xu, F., Seashore, M., Li, P. (2012) Unique genomic structure and distinct mitotic behavior of ring chromosome 21 in two unrelated cases. Cytogenet. Genome Res., 136, 180-187.
21. Doran, E., Keator, D., Head, E., Phelan, M.J., Kim, R., Totoiu, M., Barrio, J.R., Small, G.W., Potkin, S.G., Lott, I.T.(2017) Down Syndrome, Partial Trisomy 21, and Absence of Alzheimer's Disease: The Role of APP. J. Alzheimers Dis. 56, 459-470.
22. Burillo-Sanz, S., Vargas, M.T., Morales-Camacho, R.M., Caballero-Velázquez, T., Sánchez, J., García-Lozano, J.R., Pérez de Soto, I., Prats-Martín, C., Bernal, R., Pérez-Simón, J.A. (2016) RUNX1 amplification in AML with myelodysplasia-related changes and ring 21 chromosomes. Hematol. Oncol. doi: 10.1002/hon.2287

**4. References for all cases with partial trisomy 21 excluded from the study due to t(X;21) translocation**

1. Couturier, J., Dutrillaux, B., Garber, P., Raoul, O., Croquette, M.F., Fourlinnie, J.C., Maillard, E. (1979) Evidence for a correlation between late replication and autosomal gene inactivation in a familial translocation t(X;21). Hum. Genet., 49, 319-326.
2. Perez-Castillo, A., Del Mazo, J., Abrisqueta, J.A. (1983) Three interesting cases of Down's syndrome. Ann. Genet., 26, 123-128.
3. Taysi, K., Sparkes, R.S., O'Brien, T.J., Dengler, D.R. (1982) Down's syndrome phenotype and autosomal gene inactivation in a child with presumed (X;21) de novo translocation. J. Med. Genet., 19, 144-148.
